# Delayed extensive brain edema caused by the growth of a giant basilar apex aneurysm treated with basilar artery obliteration: a case report

**DOI:** 10.1186/s12883-020-01819-9

**Published:** 2020-06-06

**Authors:** Daniel García-Pérez, Irene Panero, Carla Eiriz, Luis Miguel Moreno, Pablo M. Munarriz, Igor Paredes, Alfonso Lagares, José F. Alén

**Affiliations:** Department of Neurosurgery, University Hospital 12 de Octubre, Avda de Córdoba s/n, 28041 Madrid, Spain

**Keywords:** Basilar apex aneurysm, Hunterian occlusion, Brain edema

## Abstract

**Background:**

Partially thrombosed giant aneurysms at the basilar apex (BA) artery are challenging lesions with a poor prognosis if left untreated. Here we describe a rare case of extensive brain edema after growth of a surgically treated and thrombosed giant basilar apex aneurysm.

**Case presentation:**

We performed a proximal surgical basilar artery occlusion on a 64-year-old female with a partially thrombosed giant BA aneurysm. MRI showed no ischemic lesions but showed marked edema adjacent to the aneurysm. She had a good recovery, but 3 months after surgical occlusion, her gait deteriorated together with urinary incontinence and worsening right hemiparesis. MRI showed that the aneurysm had grown and developed intramural hemorrhage, which caused extensive brain edema and obstructive hydrocephalus. She was treated by a ventriculoperitoneal shunt placement. Follow-up MRI showed progressive brain edema resolution, complete thrombosis of the lumen and shrinkage of the aneurysm. At 5 years follow-up the patient had an excellent functional outcome.

**Conclusions:**

Delayed growth of a surgically treated and thrombosed giant aneurysm from wall dissection demonstrates that discontinuity with the initial parent artery does not always prevent progressive enlargement. The development of transmural vascular connections between the intraluminal thrombus and adventitial neovascularization by the vasa vasorum on the apex of the BA seems to be a key event in delayed aneurysm growth. Extensive brain edema might translate an inflammatory edematous reaction to an abrupt enlargement of the aneurysm.

## Background

Partially thrombosed giant basilar artery (BA) aneurysms are uncommon vascular lesions with an extremely poor natural history [1]. Among such lesions, those in the upper basilar apex are the most challenging to treat, given that the aneurysm usually incorporates the normal branches of the upper BA [bilateral posterior cerebral arteries (PCAs) and superior cerebellar arteries (SCAs)], and/or vital perforating arteries [1]. So, these aneurysms pose high risks for treatment by either microsurgical aneurismal clipping or endovascular coiling [2–5]. Moreover, bypass is not effective for complex BA aneurysms [6, 7].

BA obliteration proximal to the SCA, which is known as hunterian occlusion, has been performed for such unclippable giant BA aneurysms [1, 8, 9]. It has been proposed that obliterating the direct inflow from the basilar trunk reduces the hemodynamic stress on the aneurysm, allowing for complete thrombosis of the aneurysmal sac and consequent shrinkage of the aneurysm [1, 10]. However, some cases are refractory to hunterian occlusion due to the persistence of blood flow into the aneurysms [10–13]. Perforating artery thrombosis is another problem that influences outcome [6, 7, 14, 15].

Here, we report the long-term outcome of a complex BA aneurysm treated by means of surgical occlusion of the proximal BA. Importantly, several weeks after hunterian occlusion, the patient presented at the emergency department (ED). Magnetic resonance imaging (MRI) depicted growth of the partially thrombosed giant basilar artery aneurysm by intramural hemorrhage, which caused brain compression and edema, leading to clinical deterioration.

## Case presentation

**Fig. 1:**
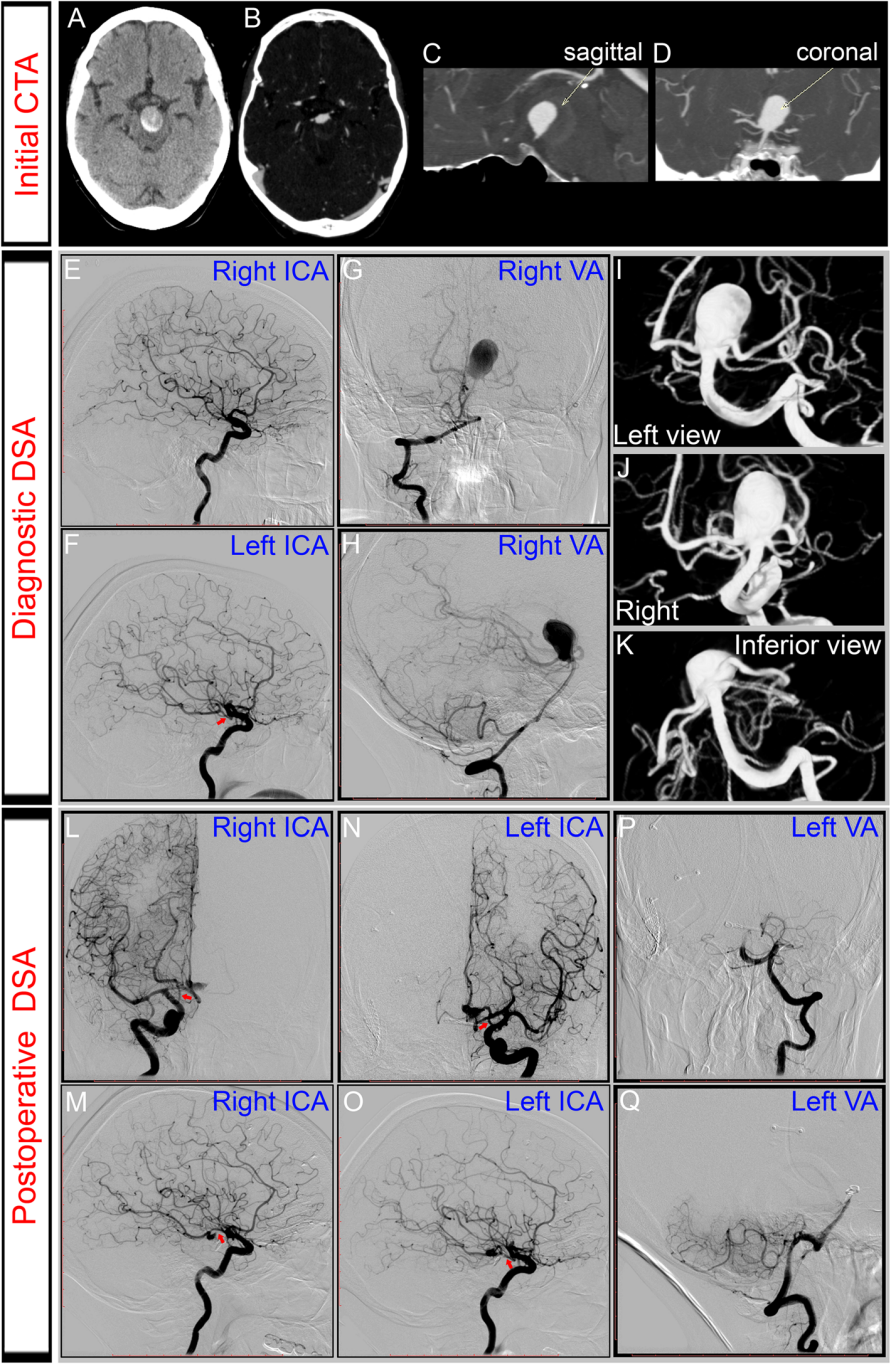
Preoperative computed tomography (CT) and digital subtraction angiography (DSA) demonstrated a basilar apex giant aneurysm occupying the interpeduncular fossa. **a**-**d**: Plain and contrast enhanced CT showed a giant calcified basilar artery aneurysm causing brain-stem compression. **e**-**k**: Diagnostic DSA with a right vertebral artery (VA) injection showing a basilar artery (BA) tip aneurysm. The diameters of the blood lumen measured 2.5 × 1.5 cm. The aneurism projected posteriorly into the interpeduncular cistern. A left robust PCoA was observed (red arrow in **f**). Based on the angiographic anatomy, trial balloon test occlusion was not required. Anteroposterior (**g**) and lateral (**h**) DSA with right VA injection showed partial filling of the aneurysm sac due to thrombosis. The aneurysm was wide-necked, with incorporation of both posterior cerebral arteries (PCAs) and superior cerebellar arteries (SCAs) into the neck (3D reconstructions, I-K). **l**-**q**: Postoperative DSA confirmed occlusion of the BA and filling of the top of the BA via the posterior communicating arteries (PCoAs) bilaterally (red arrows in L-O). DSA also revealed continued filling of the PCAs and SCAs through the PCoAs. Stagnant flow was also visible in the aneurysm, indicating a high likelihood of aneurysm thrombosis. In fact, there was a marked decrease in the diameter of the contrast filling in aneurysm blood lumen

A 64-year-old female presented with cephalea, left ptosis and diplopia. Neurological examination detected left oculomotor nerve palsy. Computed tomography (CT) revealed a partially thrombosed giant aneurysm at the tip of the BA (Fig. 1). The aneurysm projected posteriorly, significantly distorting the midbrain. An angiogram was performed, revealing a giant aneurysm at the basilar apex. Importantly, angiogram showed good collateral flow to the posterior circulation through at least one robust posterior communicating artery (PCoA).

**Fig. 2:**
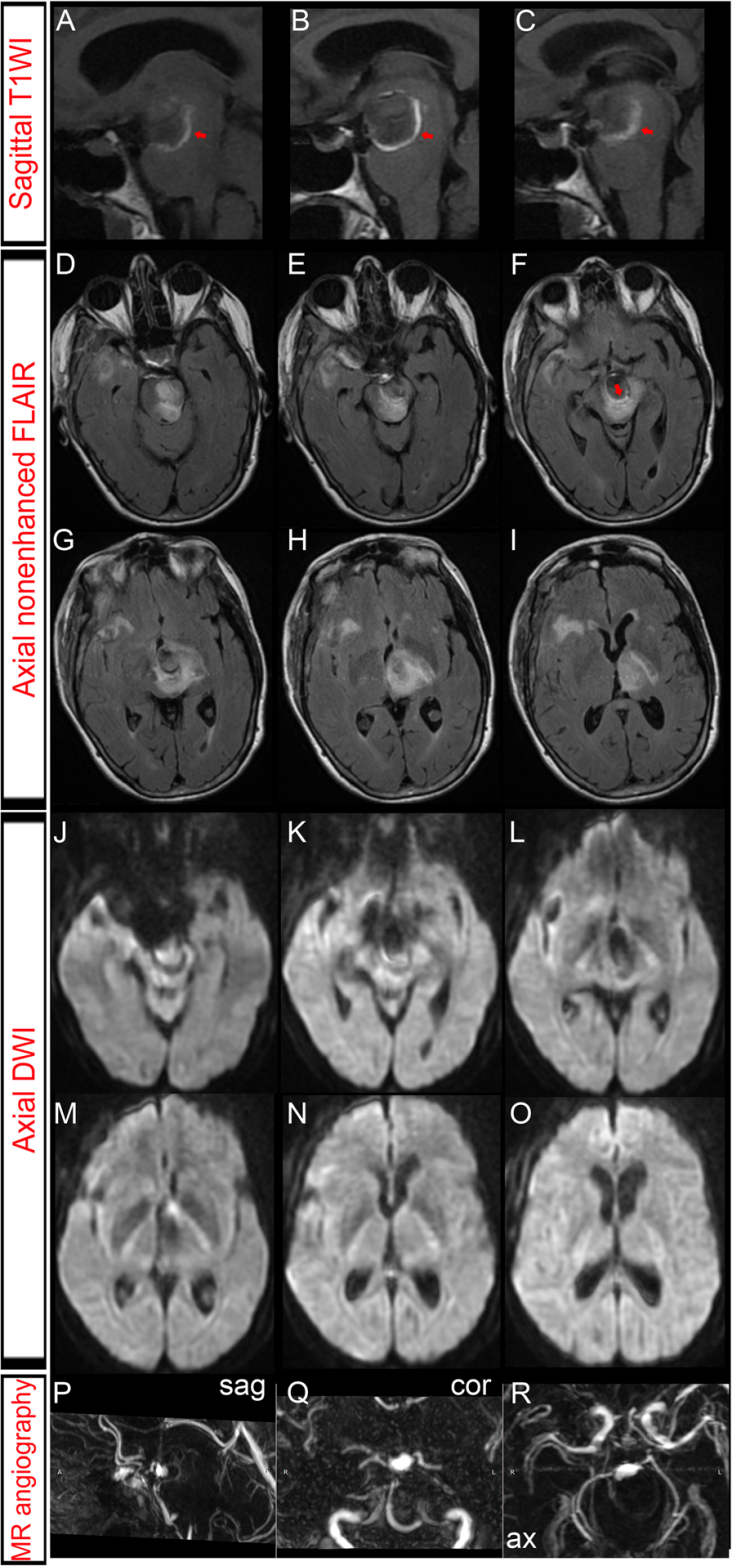
Postoperative magnetic resonance imaging (MRI) did not detect ischemic complications. **a**-**c**: Sagittal nonenhanced T1-weighted images (T1WI) exhibited a newer clot, expressed as a sickle-shaped high signal intensity area (red arrows), on the outer surface of the intraluminal old thrombus. **d**-**i**: Fluid-attenuated inversion recovery (FLAIR) MRI depicted a thrombosed giant aneurysm severely compressing the brainstem in the backward direction. In addition, we observed marked edema surrounding the basilar apex (BA) aneurysm and involving the midbrain, thalamus and left internal capsule. The sickle-shaped high signal intensity structure was also observed on FLAIR images (red arrow in F). **j**-**o**: Diffusion weighted imaging (DWI) showed no high-intensity lesions, thus excluding ischemic complications. **p**-**r**: Sagittal (sag), coronal (cor) and axial (ax) maximum intensity projection (MIP) reconstructions of magnetic resonance angiography (MRA) showed a > 50% decrease of the aneurysm blood lumen

The patient underwent BA surgical occlusion. The BA was accessed using a pterional craniotomy and a transsylvian approach. The clip placement was selected on visual inspection after identifying a perforator-free zone of the BA between the origins of the anterior inferior cerebellar arteries (AICAs) and SCAs. Electrophysiological monitoring of evoked potentials (EPs) showed no decrease of motor evoked potentials (MEPs) or somatosensory evoked potentials (SSEPs). Post-surgery angiography demonstrated stagnant flow in the dome of the aneurysm, now filling from the PCoAs (Fig. 1). Antiplatelet treatment with aspirin was initiated. Postoperatively, the patient developed a decreased level of consciousness and right hemiparesis. MRI demonstrated a marked reduction in the size of the giant aneurysm (Fig. 2). In addition, MRI showed no ischemic lesion next to the aneurysm given that no infarct was observed on diffusion-weighted MR imaging. High signal on T2-weighted images of the brain stem adjacent to the aneurysm was interpreted as edema. Over the following weeks she showed progressively increased strength and improved mentation, and was transferred to an acute rehabilitation facility. Prior to discharge, she was ambulating independently.

**Fig. 3:**
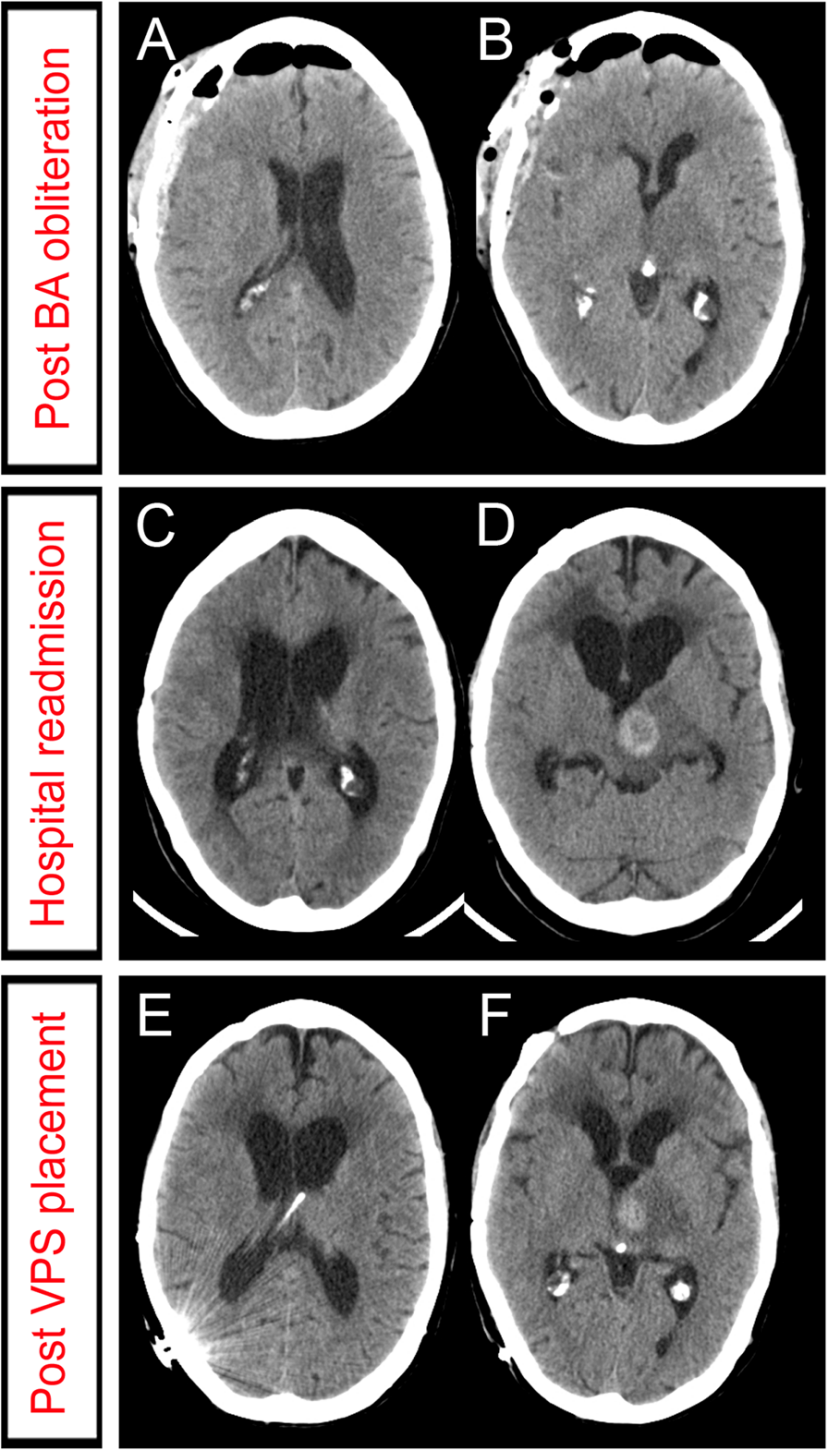
Follow up computed tomography (CT) detected a marked increase in the size of the supratentorial ventricular system together with clinical symptoms suggestive of hydrocephalus. **a**-**b**: Post-surgical changes secondary to a pterional craniotomy and a transsylvian approach, with pneumocephalus and blood remnants in the surgical site. **c**-**d**: Plain CT performed at the emergency department 3 months after surgical basilar artery occlusion. The supratentorial ventricular system was markedly increased in size compared to the previous study. We also noticed low attenuation periventricular changes around the lateral ventricles (LVs), thus suggesting transependymal edema. **e**-**f**: A right parieto-occipital ventriculoperitoneal shunt was placed, with the proximal catheter arising from the occipital horn of the right LV. Notice the decrease in the size of the supratentorial ventricular system in comparison with the previous study

**Fig. 4:**
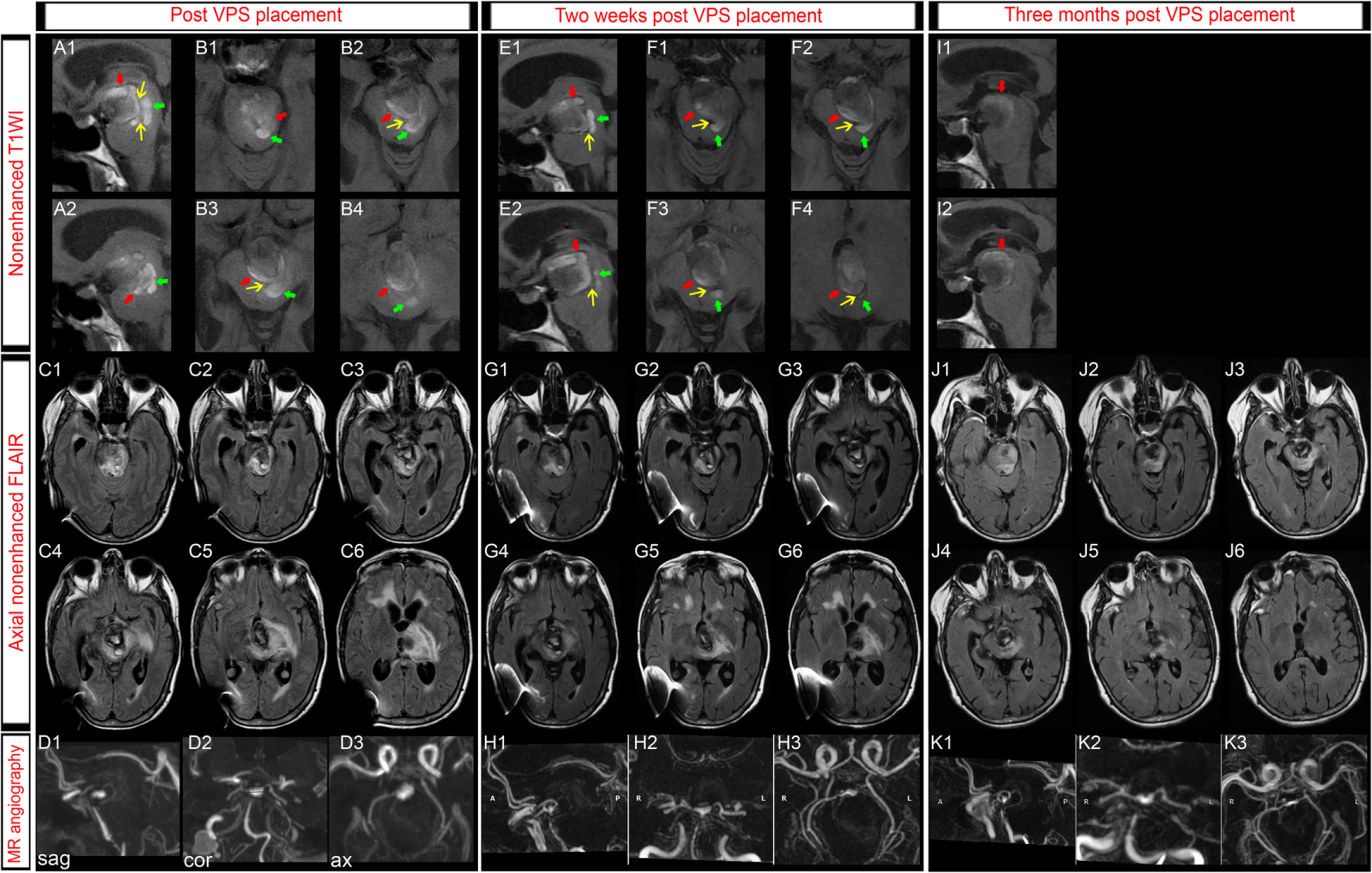
Magnetic resonance imaging (MRI) obtained on readmission to the hospital after temporary symptom relief. MRI demonstrated a large thrombosed aneurysm compressing the brainstem, with a hyperintense area indicating the presence of an acute clot within the aneurysm, besides markedly increased brain edema surrounding the enlarged aneurysm. **a**-**b**, **e**-**f**, **i**: Sagittal and axial nonenhanced T1-weighted images (T1WI) exhibited a high signal in the posterior portion of the aneurysm, suggestive of a fresh intraluminal thrombus (red arrows) as well as new intramural hematoma (green arrows; **a**-**b**), which caused a significant mass effect on the brainstem and third ventricle. The hyperintense areas on the images were located mainly on the contralateral side to the patent lumen. High signal on T1WI progressively decreased during follow up period (**e**-**f**, **i**), thus suggesting that no new hemorrhages happened. An intimal flap (yellow arrows in **a**-**b**, **e**-**f**) separated the true lumen and the false lumen. **c**, **g**, **j**: Axial fluid-attenuated inversion recovery (FLAIR) images showed a marked increase in brain edema in comparison with the previous MRI study. Intraluminal and intramural hematomas evolution were also detected on FLAIR images. **d**, **h**, **k**: Sagittal (sag), coronal (cor) and axial (ax) maximum intensity projection (MIP) reconstructions of magnetic resonance angiography (MRA) confirmed progressive thrombosis and reduced contrast filling in the residual blood lumen of the giant basilar apex aneurysm

Three months after BA occlusion, she was referred to our institution because of progressive gait instability, urinary incontinence, worsening right hemiparesis, binge eating behaviour and memory difficulties. Plain CT scan demonstrated ventricular dilatation (Fig. 3). MRI showed high signal at the periphery of the aneurysm on T1-weighted imaging, which was interpreted as a fresh intramural clot (Fig. 4). Aneurysm dissection caused severe brain-stem compression and obstructive hydrocephalus. Increased brain edema was also evident on MR images. Obstructive hydrocephalus was treated by a ventriculoperitoneal shunt placement.

**Fig. 5:**
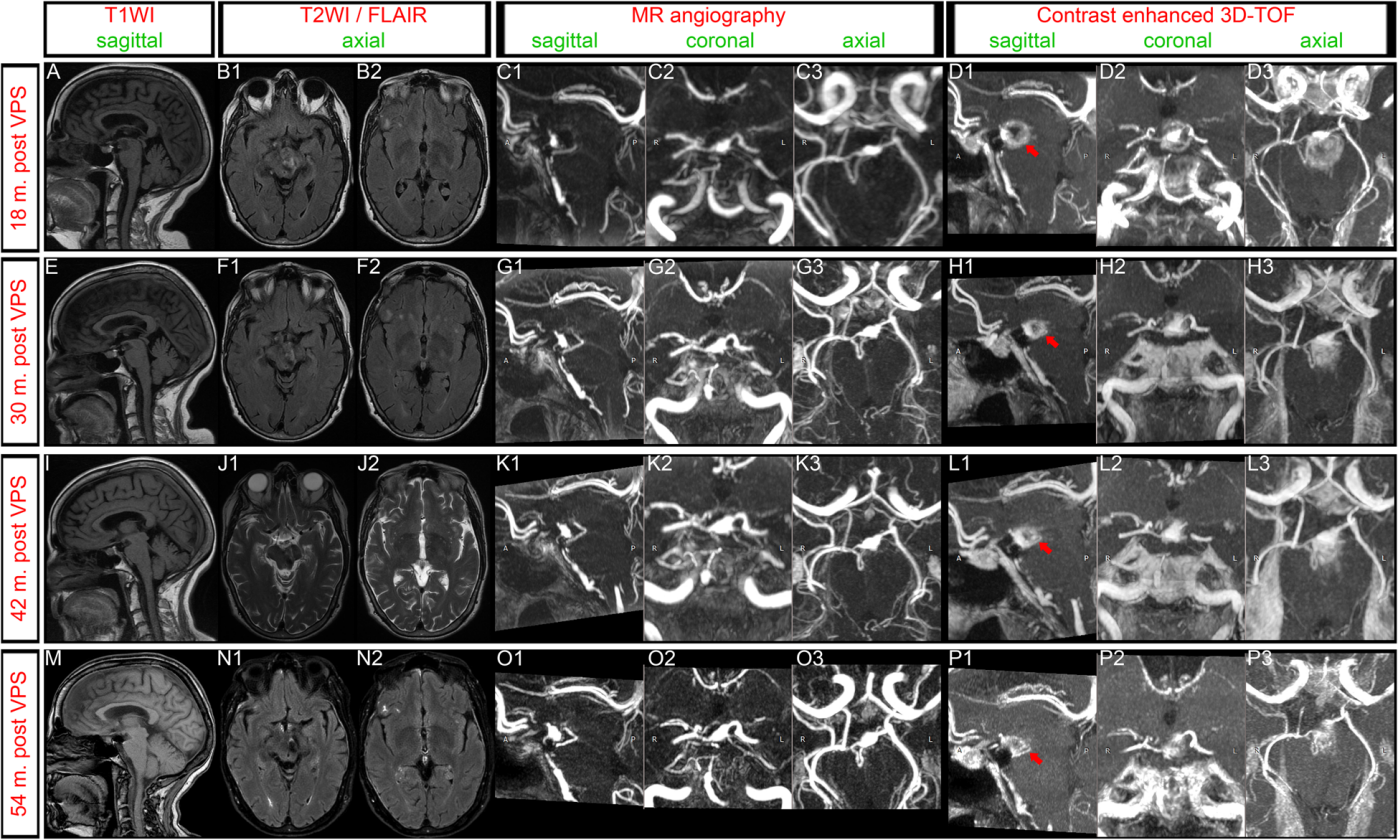
Follow-up magnetic resonance imaging (MRI) showed almost complete thrombosis of the lumen of the aneurysm and progressive reduction of the aneurysmal external contour. **a**, **e**, **i**, **m**: Sagittal T1-weighted images (T1WI) showed resolution of the intramural hematoma, no new intraluminal fresh thrombus and progressive decrease in the maximum external diameter of the aneurysm, thus alleviating brainstem compression. **b**, **f**, **j**, **n**: Axial fluid-attenuated inversion recovery (FLAIR) and/or T2-weighted images (T2WI) showed progressive resolution of brain edema. **c**, **g**, **k**, **o**: Sagittal, coronal and axial maximum intensity projection (MIP) reconstructions of magnetic resonance angiography (MRA) showed no filling of the aneurysm sac, and filling of the upper basilar apex via both posterior communicating arteries (PCoAs). **d**, **h**, **l**, **p**: Sagittal, coronal and axial MIP reconstructions of contrast enhanced 3-dimensional time of flight (3D-TOF). Although no filling of the aneurysm was found, a marked gadolinium enhancement is noted around the top of the basilar artery, representing the old intraluminal thrombus. The maximum external diameter of gadolinium enhancement decreased over the follow-up period (red arrows)

Long-term follow-up has extended for 5 years. The patient has made an excellent recovery and the hemiparesis has almost resolved. Left oculomotor palsy was completely recovered. Follow-up MR imaging demonstrated a marked reduction in the size of the giant aneurysm with no filling of the aneurysm sac, and filling of the upper BA via the PCoAs (Fig. 5). Intramural hematoma resolved and brain edema markedly diminished.

## Discussion

### Surgical alternatives for thrombosed giant upper BA aneurysm

In the present report, we assessed the radiological and neurological results of a partially thrombosed giant upper BA aneurysm treated with BA proximal occlusion.

Microsurgical aneurysmal clipping or endovascular coiling of partially thrombosed giant BA aneurysms result in high complication rates [16]. Proximal BA occlusion is safer and more effective than other therapies [9]. The aim of the surgery is the occlusion of the BA without impairment of brainstem perforators, while maintaining collateral blood flow to the posterior circulation by PCoAs. We performed a right pterional transsylvian approach. Advantages of the transsylvian approach are its widespread familiarity among neurosurgeons, less temporal lobe retraction, potential for visualization of the opposite P1, and larger surgical field compared with the subtemporal approach [17]. When a choice is possible, the senior author usually prefers the right side approach. It reduces the risk of injury of the dominant lobe and facilitates clipping for right handed surgeons, which is the case. Although the patient presented a left oculomotor palsy, the objective of the surgery was not to decompress the third cranial nerve, but only find a safe region to place a clip in the basilar trunk.

### Early complication following hunterian occlusion

Flow redirection to the BA apex from the anterior circulation is supposed to reduce the hemodynamic burden and to promote intra-aneurysmal thrombosis, thus reducing the size of the aneurysm [1, 8, 9]. In agreement, a few days after surgery, digital subtraction angiography (DSA) and MR angiography showed a markedly reduced intra-aneurysmal flow. However, our patient developed a decreased level of consciousness and right hemiparesis following hunterian occlusion. By far, the most common cause of surgical morbidity of the aneurysms treated with BA occlusion is infarction of the perforating arteries territory, which supplies the mesencephalon as well as the hypothalamus and thalamus [6, 7, 14, 15]. Surgical occlusion of thalamoperforators could even impair the so-called rostroreticular activation system and lead to coma or somnolence. So, all of the thalamoperforators should be preserved during neurosurgical procedures regardless of their caliber [18]. Accordingly, our clip placement was selected on visual inspection of a perforator-free zone to ensure safe clip application [9]. However, perforator territory infarction (PTI) can occur even without direct injury of the parent artery or occlusion of perforators [14]. PTI is usually diagnosed on diffusion-weighted imaging (DWI). Larger aneurysm size, basilar artery aneurysms and localization in the interpeduncular cistern (IPC) are significantly related to PTI, which has a negative impact on outcome [6, 7, 14, 15]. Postoperative DWI revealed neither ischemic nor hemorrhagic complications. So, no ischemic complication associated with insufficient perfusion caused by BA obliteration seemed to be related with the clinical worsening in our patient. Consequently, the patient’s motor deficit was attributed to aneurysm thrombosis, increased brain edema and mass effect. Therefore, the patient gradually improved as brain edema progressively resolved and the aneurysm reduced in size.

### Delayed complication following hunterian occlusion

Several weeks after hunterian occlusion, our patient presented with clinical deterioration and radiological obstructive hydrocephalus. The occurrence of obstructive hydrocephalus due to an intracranial aneurysm is rare, whereas the basilar bifurcation is their most common location [19–22]. Neuroimaging findings demonstrated a stable reduced aneurysm lumen but a new intramural hemorrhage (IMH) which caused aneurysmal growth and severe midbrain compression. Coexistence of luminal narrowing and aneurysmal external contour dilatation have been considered helpful in detecting aneurysm dissection [23]. In the present report, we had switched the parent artery from the basilar artery to the PCoAs. Thus, it could be argued that the changes in the direction and the diameter of the parent arteries might have resulted in a focal elevation of wall shear stress on the posterior aneurysm wall, and that these hemodynamic changes might have been associated with the subsequent wall dissection [24]. Nevertheless, BA ligation has been described to reduce and change the flow of an aneurysm, allowing complete organization of the thrombus and avoiding hemodynamic injury to the aneurysm wall [10]. So, it seems that surgical intervention can stabilize several aneurysms, but can cause rupture in others. In this sense, preoperative computational flow dynamics simulation might help to predict postoperative intraluminal flow and so, help to establish the best strategy [24]. However, we consider that increased wall shear stress due to hemodynamic changes was unlikely to be the mechanism that enabled IMH because the hemorrhage occurred at the periphery of the aneurysm wall within the clot, but far from the patent lumen, as previously documented [25].

### Pathophysiological mechanisms underlying thrombosed aneurysm enlargement

Literature supports persistent growth of completely thrombosed giant aneurysms [26–29]. Different pathomechanisms have been reported. On the one hand, intrathrombotic vascular channels development and consequent intrathrombotic hemorrhages have been proposed [30]. On the other hand, growth of these giant aneurysms might be attributable to neovascularization and recurrent intramural hemorrhages [25, 26, 28, 31]. As shown in Fig. 4, we observed that the thrombosed portion grew due to both, hemorrhage into the thrombus and in the aneurismal wall. IMH is considered to be caused by the rupture of the vasa vasorum or newly formed wall vessels [29, 32]. The strong peripheral contrast enhancement that we observed (Fig. 5) could be attributed to the highly vascularized arterial wall [26]. Numerous factors are thought to trigger neovascularization, such as an intra-aneurysmal thrombus [33]. In fact, most growing intracranial giant aneurysms contain a thrombus within the aneurysm sac [34]. Our case supports this concept, and suggests that the rapid formation of an intra-aneurysmal thrombus following BA occlusion may have promoted mural damage, helping to trigger neovascularization and the growth of capillaries. Our report suggests that the thrombotic process started right after the surgical proximal occlusion and not at distance. This would also explain the significant edema and worsening of clinical condition that developed following BA occlusion. Delayed worsening of the thrombosis and new IMH would have led to hydrocephalus and a new neurological worsening (supplementary Figure 1). We conclude that neovascularization processes and repeated minor hemorrhages into the aneurysm thrombus and wall led to the delayed increased mass effect, increased edema and consequently, deterioration in our patient’s neurological condition.

Very few reports describe giant aneurysms presenting with massive cerebral edema and clinical deterioration [35, 36]. The authors proposed that an abruptly enlarged aneurysm compromised blood supply to the adjacent brain tissue and caused ischemia and subsequent edema due to a loss of vasoresponsivity and breakdown of the blood-brain barrier [35, 36]. The present case presented with extensive edema following aneurysm growth, but no ischemic lesions next to the aneurysm were observed on DWI. Nakayama and colleagues reported a case of spontaneous rapid growth of a partially thrombosed giant basilar artery aneurysm caused by intramural hemorrhage. With conservative treatment, her ataxia and urinary incontinence resolved [36]. In contrast, Heros and Kolluri reported two cases of giant left middle cerebral artery aneurysm presenting with rapidly progressing hemiparesis and aphasia. CT showed recent intraaneurysmal thrombosis and massive edema and swelling of the cerebral hemisphere. In both patients, surgical resection of the aneurysm was accomplished, but the outcome was disastrous [35]. Alternatively, the enhancing wall and the edematous reaction of the adjacent brain parenchyma might be a sign for an inflammatory pathomechanism [25, 37, 38], although the role of vasa vasorum concerning the rim enhancement might also account for these findings. Medical, i.e. anti-inflammatory treatment (such as steroids), could have been proposed given the likelihood of an inflammatory pathomechanism (either causative or reactional).

IMHs have the same signal evolution on MRI, and signals are likely to appear isointense or unrecognizable within 2 months [39, 40]. The resolution of intramural hemorrhagic signals in our case indicate cessation of intramural hemorrhage. During the long-term follow-up, we have also observed progressive brain edema resolution, decrease in the residual blood lumen and marked shrinkage of the aneurysm, together with improvement of the neurological condition, highlighting that thrombosis and shrinkage might be dynamic processes. We propose that, once hemodynamic factors following hunterian ligation had been stabilized, block of the blood flow to intrathrombotic vascular channels promoted absorption of the organized thrombus and shrinkage of the aneurysm.

## Conclusions

Growth of a surgically treated and thrombosed giant aneurysm demonstrates that discontinuity with the initial parent artery does not always prevent progressive enlargement. The development of transmural vascular connections between the intraluminal thrombus and neovascularized adventitial wall appears to be a key event in delayed aneurysm growth. Extensive brain edema might translate an inflammatory edematous reaction to abrupt enlargement of the aneurysm. Conservative management is recommended.

## Supplementary information

**Additional file 1 **Figure S1. Comparison between postoperative magnetic resonance imaging (MRI) and a new MRI obtained on readmission to the hospital after temporary symptom relief. **A-C:** Sagittal nonenhanced T1-weighted images (T1WI) exhibited a newer clot, expressed as a sickle-shaped high signal intensity area, on the outer surface of the intraluminal old thrombus. **D-I:** Fluid-attenuated inversion recovery (FLAIR) MRI depicted a thrombosed giant aneurysm severely compressing the brainstem in the backward direction. In addition, we observed marked edema surrounding the basilar apex (BA) aneurysm and involving the midbrain, thalamus and left internal capsule. The sickle-shaped high signal intensity structure was also observed on FLAIR images. **J:** Axial maximum intensity projection (MIP) reconstructions of magnetic resonance angiography (MRA) showed a decrease of the aneurysm blood lumen. **K:** Axial MIP reconstructions of contrast enhanced 3-dimensional time of flight (3D-TOF). A marked gadolinium enhancement is noted around the aneurysm, representing the new intraluminal thrombus. **L-N:** Sagittal nonenhanced T1WI exhibited a high signal in the posterior portion of the aneurysm, suggestive of a fresh intraluminal thrombus as well as new intramural hematoma, which caused a significant mass effect on the brainstem and third ventricle. **O-T:** Axial FLAIR images showed a marked increase in brain edema in comparison with the previous MRI study. Intraluminal and intramural hematomas evolution were also detected on FLAIR images. **U:** Axial MIP reconstructions of MRA confirmed reduced contrast filling of the blood lumen of the giant basilar apex aneurysm. **V:** Axial MIP reconstructions of 3D-TOF showed gadolinium enhancement around the aneurysm, representing the intraluminal and intamural thrombus.

## Data Availability

Additional data and material would be made available upon request.

## Abbreviations

AICA: Anterior inferior cerebellar artery
BA: Basilar artery
CT: Computed tomography
DWI: Diffusion-weighted imaging
DSA: Digital subtraction angiography
ED: Emergency department
EPs: Evoked potentials
IPC: Interpeduncular cistern
IMH: Intramural hemorrhage
MRI: Magnetic resonance imaging
MEPs: Motor evoked potentials
PTI: Perforator territory infarction
PCA: Posterior cerebral artery
PCoA: Posterior communicating artery
SSEPs: Somatosensory evoked potentials
SCA: Superior cerebellar artery
