# Supplementary figures and images for: Delayed extensive brain edema caused by the growth of a giant basilar apex aneurysm treated with basilar artery obliteration: a case report
Source: BMC Neurol. 2020 Jun 6;20:232. doi: 10.1186/s12883-020-01819-9 (PMC7275367; doi:10.1186/s12883-020-01819-9)

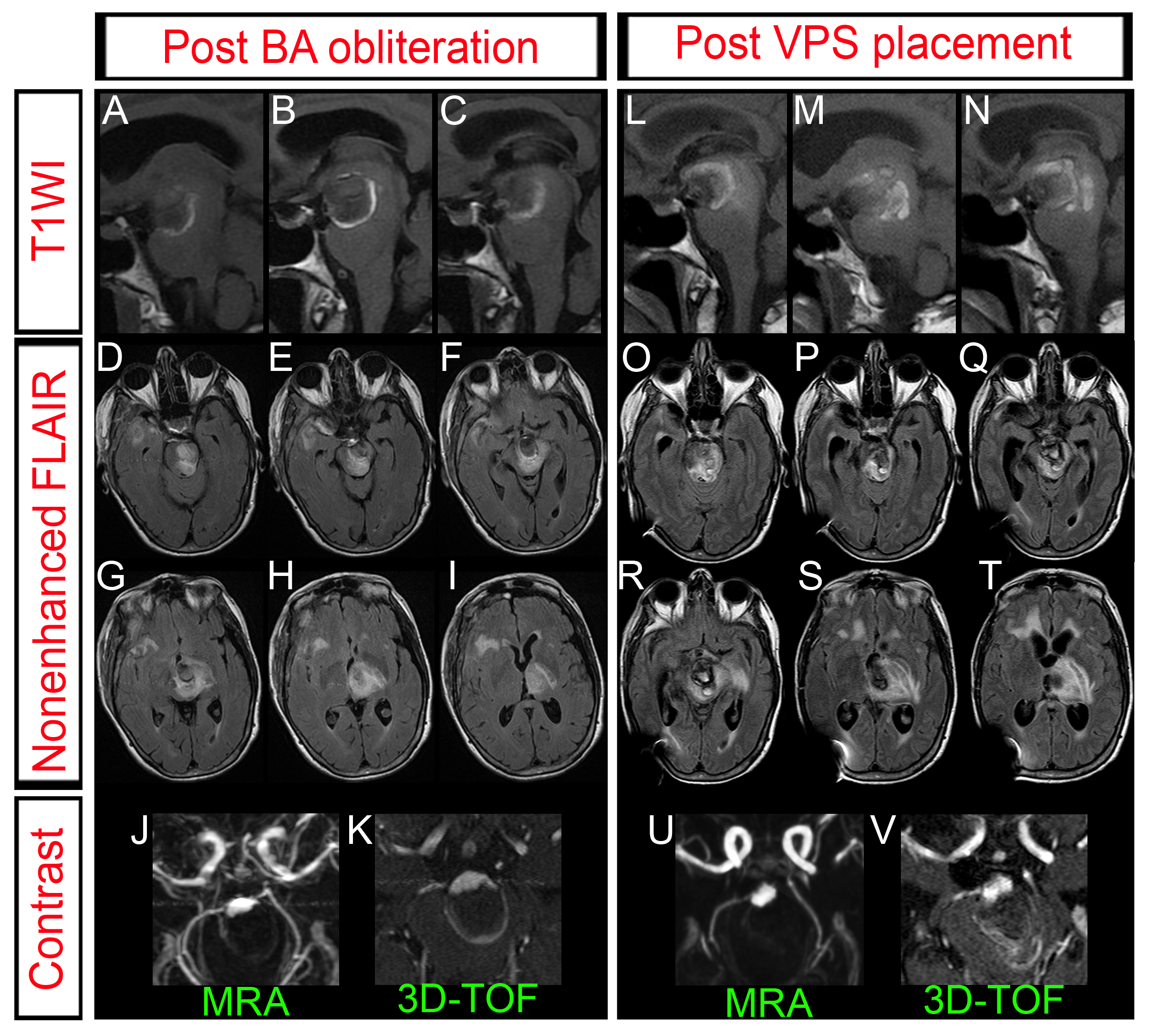

Supplement: Supplementary file 1 — Additional file 1 Figure S1. Comparison between postoperative magnetic resonance imaging (MRI) and a new MRI obtained on readmission to the hospital after temporary symptom relief. A-C: Sagittal nonenhanced T1-weighted images (T1WI) exhibited a newer clot, expressed as a sickle-shaped high signal intensity area, on the outer surface of the intraluminal old thrombus. D-I: Fluid-attenuated inversion recovery (FLAIR) MRI depicted a thrombosed giant aneurysm severely compressing the brainstem in the backward direction. In addition, we observed marked edema surrounding the basilar apex (BA) aneurysm and involving the midbrain, thalamus and left internal capsule. The sickle-shaped high signal intensity structure was also observed on FLAIR images. J: Axial maximum intensity projection (MIP) reconstructions of magnetic resonance angiography (MRA) showed a decrease of the aneurysm blood lumen. K: Axial MIP reconstructions of contrast enhanced 3-dimensional time of flight (3D-TOF). A marked gadolinium enhancement is noted around the aneurysm, representing the new intraluminal thrombus. L-N: Sagittal nonenhanced T1WI exhibited a high signal in the posterior portion of the aneurysm, suggestive of a fresh intraluminal thrombus as well as new intramural hematoma, which caused a significant mass effect on the brainstem and third ventricle. O-T: Axial FLAIR images showed a marked increase in brain edema in comparison with the previous MRI study. Intraluminal and intramural hematomas evolution were also detected on FLAIR images. U: Axial MIP reconstructions of MRA confirmed reduced contrast filling of the blood lumen of the giant basilar apex aneurysm. V: Axial MIP reconstructions of 3D-TOF showed gadolinium enhancement around the aneurysm, representing the intraluminal and intamural thrombus. [file 12883_2020_1819_MOESM1_ESM.tif]
